# Eco-Friendly Preparation of a Polyimide/Polyethylene Composite Separator and Its Application in Lithium-Ion Batteries

**DOI:** 10.3390/polym18151871

**Published:** 2026-07-30

**Authors:** Hyun-Soo An, Yun-Je Choi, Dam-Bi Kim, Yasaswini Oruganti, Dae-Woon Lim, Seungwon Song, Woojun Choi, Chan-Moon Chung

**Affiliations:** 1 Department of Chemistry, Yonsei University, Wonju 26493, Republic of Korea; gustn2633@naver.com (H.-S.A.); qqldkkr95@gmail.com (D.-B.K.); oyasaswini@yonsei.ac.kr (Y.O.); limdaewoon@yonsei.ac.kr (D.-W.L.); 2 Xerabrid Corporation, Hwaseong 18469, Republic of Korea

**Keywords:** lithium-ion battery, composite separator, polyimide, eco-friendly preparation

## Abstract

The surface modification of polyolefin separators for lithium-ion batteries using polymer particles has been extensively investigated to enhance their electrolyte wettability, thermal resistance, and mechanical properties. However, the traditional polymer synthesis and/or polymer-based separator modifications have mostly been carried out using harmful and expensive organic solvents. In this study, a powder-type polyimide (PI) was synthesized using water as a solvent, and then PI-particle-containing coating slurries were prepared in an aqueous dispersion medium. The coating slurries were applied on a polyethylene (PE) separator to obtain PI-particle-coated PE (PI-PE) separators. Thermal and mechanical properties were evaluated for the PI-PE separators. Electrolyte uptake, porosity, air permeability, and ionic conductivity of the separators were also evaluated. The electrochemical properties of coin cells assembled with the PI-PE separator were evaluated by charge–discharge property. Coating of PI particles improves the thermal stability and electrolyte wettability of the PE separator, and LiCoO_2_/PI-PE separator/Li half-cells showed battery performance similar to that of bare PE half-cells. This work offers insights into the simple, eco-friendly preparation of a separator with excellent thermal stability, electrolyte wettability and effective ionic conductivity.

## 1. Introduction

Lithium-ion batteries (LIBs) are the leading energy storage technology in portable electronics, electric vehicles, and grid applications due to their high energy density and long cycle life [1,2]. Within an LIB, the separator is an electrically insulating but ion-permeable membrane that prevents direct contact between the cathode and anode while enabling ionic conduction. Although electrochemically inert, the separator strongly affects the performance, safety, and lifetime of the cell. Therefore, the development of advanced separators is essential for the increasing demands of next-generation LIBs.

Commercial separators are typically fabricated from polyolefin materials such as polyethylene (PE) and polypropylene (PP). These membranes exhibit desirable chemical stability and mechanical strength at room temperature. However, they suffer critical drawbacks, including poor electrolyte wettability due to their intrinsic hydrophobicity and low thermal resistance, which can cause severe shrinkage at temperatures greater than 120 °C [3,4]. Such limitations compromise both electrochemical performance and safety, highlighting the urgent need for improved separator technologies.

To address these challenges, surface modification of polyolefin membranes with ceramic or polymer particles has been investigated [5,6,7,8]. Ceramic coatings such as Al_2_O_3_ and SiO_2_ can enhance thermal stability and safety [9,10,11,12], but they also increase the weight of inactive components, reduce mechanical flexibility, and are prone to interfacial cracking under stress [13,14,15]. In contrast, polymer coatings offer several advantages, including lower inactive mass, better crack resistance, improved process compatibility, facile scale-up to large areas, and superior industrial applicability [15,16,17]. A variety of polymers has been employed as coating materials, including poly(vinylidene fluoride) (PVDF), polyimide (PI), sulfonated poly(arylene ether ketone) (SPAEK), ethylene–vinyl alcohol copolymer (EVOH), polydopamine (PDA), aramid nanofibers, poly(ε-caprolactone) (PCL), and poly(methyl methacrylate) (PMMA) [18,19,20,21,22,23,24,25,26]. Among them, PI has drawn particular attention owing to its exceptional thermal stability, mechanical robustness, and favorable compatibility with liquid electrolytes. These attributes make PI a suitable candidate for separator modification to improve both safety and electrochemical properties. However, despite the advantages of polymer coatings for separators, most polymer coating processes reported to date rely on organic solvents such as *N*-methyl-2-pyrrolidone (NMP), dimethylformamide (DMF), dimethylacetamide (DMAc), and methanol during polymer synthesis and/or during coating slurry preparation. The use of toxic and costly solvents poses environmental and health hazards and increases manufacturing complexity. Consequently, there is a strong motivation to develop water-based polymer coating technologies that can eliminate harmful solvents while maintaining or improving separator performance.

In this study, we report a water-based synthesis and coating approach to deposit PI particles onto PE separators. The resulting composite membranes exhibit significantly enhanced thermal stability and improved electrolyte wettability, demonstrating the feasibility of eco-friendly polymer particle coatings. This work contributes to the sustainable manufacturing of advanced polyolefin separators and demonstrates the potential of eco-friendly polymer-coated separators for battery applications.

## 2. Materials and Methods

### 2.1. Materials

Pyromellitic dianhydride (PMDA), 4,4′-oxydianiline (ODA), carboxymethyl cellulose (CMC, *n* = approx. 1050), a mixture of 1 M lithium hexafluorophosphate (LiPF_6_) in ethylene carbonate/diethylene carbonate (EC/DEC, 1/1, *v*/*v*), and fluoroethylene carbonate (FEC) were purchased from Merck-Korea (Seoul, Republic of Korea). All used water was double distilled. NMP and *n*-butanol (99%) were purchased from Duksan Pure Chemical (Seoul, Republic of Korea) and were used without further purification. Pyridine was purchased from Samchun Chemicals (Seoul, Republic of Korea) and acetic anhydride was purchased from Junsei Chemical (Tokyo, Japan). A commercial PE (Bare PE) separator was purchased from SK Innovation (thickness = 12 μm, Seoul, Republic of Korea). Spherical Al_2_O_3_ (average size = 500 nm) and SiO_2_ (average size = 8 nm) powders were purchased from Denka Co. (Tokyo, Japan) and RND Korea Co. (Gwangmyeong, Republic of Korea), respectively.

### 2.2. Preparation of PI-Coated Separators

PMDA (10.9 g, 50 mmol) was completely dissolved in water (250 g) under nitrogen atmosphere at 60 °C, and ODA (10.0 g, 50 mmol) was added. Pyridine (7.91 g, 100 mmol) and acetic anhydride (10.2 g, 100 mmol) were added, and the resultant suspension was stirred under nitrogen atmosphere at 100 °C for 24 h. After the reaction was completed, the mixture was poured into water and acetone was added. A yellow color PI powder was obtained by filtration followed by washing with water. The PI powders were dried at 40 °C for 12 h in a vacuum.

A coating slurry was prepared by mixing the PI powder and CMC in water. After the PI powder and CMC were put into water, the mixture was subjected to ultrasonication under 20 kHz and an amplitude of 20% at room temperature for 5 min (VCX750, Sonicx & Materials, Inc., Newtown, CT, USA). The weight of the PI powder was fixed at 15 wt%, and the contents of the CMC binder were adjusted at 0.1 wt%, 0.3 wt%, and 0.5 wt% (PI-PE(0.1CMC) separator, PI-PE(0.3CMC) separator, and PI-PE(0.5CMC) separator, respectively). The prepared coating slurries were coated on one side of Bare PE separator by simple doctor blade coating process. The coated separators were dried at room temperature (25 °C) and further dried at 50 °C for 12 h in a vacuum.

### 2.3. Characterization

The synthesized PI powder was identified using Fourier transform infrared (FT-IR) spectroscopy (Spectrum One B, Perkin Elmer Co., Waltham, MA, USA) in the wavenumber range of 400–4000 cm^−1^. Thermal degradation properties of the PI were studied by thermogravimetric analysis (TGA, TGA-55, TA instrument, New Castle, DE, USA). The TGA tests were carried out under a N_2_ atmosphere at a heating rate of 10 °C/min and the temperature was increased from 30 °C to 800 °C. Differential scanning calorimetry (DSC) of the PI was conducted under a N_2_ atmosphere at a heating rate of 10 °C/min and the temperature was increased from 30 °C to 400 °C. Since the synthesized PI was not soluble in organic solvents, its inherent viscosity was measured in a concentrated sulfuric acid at a concentration of 0.5 g/dL. The surface morphology of the separators was observed using a field emission scanning electron microscope (FE-SEM, JSM-7900F, JEOL, Tokyo, Japan) at an accelerating voltage of 5 kV and working distance of 6 mm. The thickness and morphology of cross-section of the separators were also observed using the FE-SEM at an accelerating voltage of 5 kV and working distance range of 7.5–8.5 mm. Water contact angles were measured using a contact angle analyzer (DSA-25, KRUSS, Gmbh, Germany) by sessile drop method with deionized water. The electrolyte wettability of the separators was examined by comparing the electrolyte wetting area of each separator at room temperature, which had the same size (2 × 2 cm^2^). The electrolyte uptake amount of the separators was calculated by Equation (1),

Electrolyte uptake (%) = (W − W_0_)/W_0_ × 100 (1)

where W_0_ and W represent the weights of the separators before and after absorbing electrolyte at room temperature for 1 h, respectively.

The porosity of the separators was measured using the *n*-butanol method at room temperature for 1 h, and calculated by Equation (2),

Porosity (%) = (W_butanol_/ρ_butanol_)/{(W_separator_/ρ_separator_) + (W_butanol_/ρ_butanol_)} (2)

where W_separator_ represents the weight of the separator before soaking and W_butanol_ represents the weight of absorbed *n*-butanol after soaking. ρ_separator_ and ρ_butanol_ represent the density values of separator and *n*-butanol at room temperature, respectively. The air permeability of the separators was measured using by a Gurley densometer (KP-M4350, Kipae E&T Co., Ltd., Seoul, Republic of Korea) to measure the time for 100 mL air to pass through under room temperature and pressure. The effective ionic conductivity of the separators was measured using electrochemical impedance spectroscopy (electrochemical workstation SI-1260, Solartron Analytical, Bognor Regis, UK). The 2032 coin-type cells were assembled by sandwiching the separator between two stainless-steel (SS) electrodes of 1.77 cm^2^ in area and soaking it into the liquid electrolyte for ionic conductivity measurement. The AC impedance measurement was carried out under the frequency range of 0.01 Hz to 1 MHz with the amplitude of 100 mV at room temperature. The effective ionic conductivity (σ_eff_) was calculated by Equation (3),

Effective ionic conductivity (σ_eff_) = *d*/*R_b_A* (3)

where *d* is the thickness of the separator, *A* is the area of the stainless-steel electrode, and *R_b_* is the bulk resistance measured by AC impedance. Mechanical strength measurements of the separators was carried out to measure the tensile and puncture strength using a universal testing machine (UTM, WL2100C, Withlab Co., Gunpo-si, Republic of Korea) at room temperature. Thermal shrinkage of the separators was measured from the dimensional changes after heat exposure at 140 °C for 1 h, and was calculated by Equation (4),

Thermal shrinkage (%) = (S_0_ − S)/S_0_ × 100 (4)

where S_0_ and S represent the areas of the separators before and after the heat exposure, respectively. Thermal stability of the separators was identified using by DSC under a N_2_ atmosphere at a heating rate of 10 °C/min and the temperature was increased from room temperature to 300 °C.

### 2.4. Evaluation of Electrochemical Performance of Cells

The 2032 coin-type half-cells were assembled by sandwiching the separator between the LiCoO_2_ (LCO) cathode and a lithium metal anode (thickness = 300 μm) and filling with liquid electrolyte (1 M LiPF_6_ in EC/DEC/FEC, 5/5/1, *v*/*v*/*v*). The whole assembly process was carried out in an Ar-filled glove box. The cathode was prepared by coating the NMP-based slurry, which was composed of 92 wt% of LiCoO_2_ (L&F Co., Daegu, Republic of Korea), 4 wt% of acetylene black (Super-P), and 4 wt% of PVDF (Kynar 2801, Arkema Co., Puteaux, France), on aluminum foil. The cast foils were dried at 110 °C for 6 h in a vacuum. The electrochemical performance of the cells was conducted to evaluate the discharge capacity, C-rate capability, and cycling performance using a charge–discharge cycle tester (ZIVE MP, ZIVELAB, Seoul, Republic of Korea) at room temperature. The discharge capacity of the cells was measured by cycling at a charge–discharge rate of 0.5 C at a voltage range of 2.5–5 V. The C-rate capability was identified in cells by cycling at a charging rate of 0.2 C and a discharging rate of 0.2 C, 0.5 C, 1 C, 2 C, and 5 C in the voltage range of 3–4.3 V. The cycling performance of the cells was evaluated under constant current mode over a voltage range of 3–4.3 V by cycling at a charging and a discharging rate of 1 C.

## 3. Results and Discussion

### 3.1. Preparation and Characterization of PI-PE Separators

**Figure 1:**
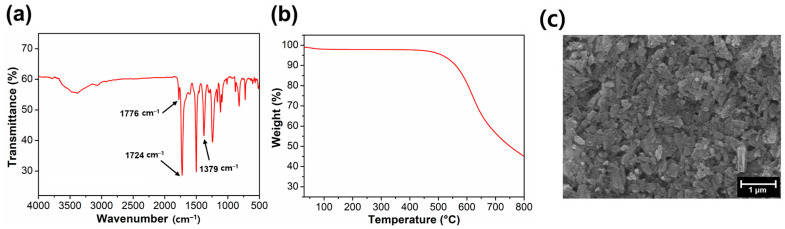
(**a**) FT-IR spectrum, (**b**) TGA curve, and (**c**) SEM image of PMDA-ODA PI.

A powder-type PI was synthesized by reaction of pyromellitic dianhydride (PMDA) and 4,4′-oxydianiline (ODA) in water as a solvent in the presence of pyridine catalyst [27,28] (Scheme S1). As shown in Figure 1a, formation of the PI was confirmed by Fourier transform infrared (FT-IR) spectroscopy. Absorption bands due to asymmetric and symmetric stretching of C=O in imide rings were observed at 1776 cm^−1^ and 1724 cm^−1^, respectively. Also, the absorption band of imide C—N stretching was observed at 1379 cm^−1^. Thermal properties of the PMDA-ODA PI powder were measured by thermogravimetric analysis (TGA) under a N_2_ atmosphere. Figure 1b shows that the PI powder retained its weight up to about 500 °C: it had a T_5_ of 513 °C and T_10_ of 560 °C. Differential scanning calorimetry (DSC) observed no glass transition for the PMDA-ODA PI, indicating that the PI powder synthesized in water is a suitable coating material to improve the thermal stability of polyolefin separator. As observed in the field emission scanning electron microscope (FE-SEM) image of the PI powder in Figure 1c, the particles had irregular shapes with an average size of approximately 390 nm, although a broad size distribution ranging from about 100 to 900 nm was observed. This size non-uniformity is expected to promote dense packing during composite formation, potentially contributing to the formation of a robust surface layer. In addition, the inherent viscosity of the PI was measured to be 0.21 dL/g (the molecular weight of the PI could not be measured because of its insolubility in organic solvents). Since the PI was oligomeric, many end groups (carboxyl and amino groups) may exist on the particle surface, contributing to a hydrophilic surface [27].

To elucidate the dispersion stability of PMDA-ODA PI powder, a 15-wt% PI dispersion in water was prepared by ultrasonication (30 kHz) for 5 min (Figure S1). Similarly, 15-wt% dispersions containing Al_2_O_3_ and SiO_2_ powders were prepared for comparison. After storage for 24 h, the PI particles remained stably dispersed, while Al_2_O_3_ and SiO_2_ particles settled at the bottom of the vials. This indicates that the PI powder has good dispersion stability without aggregation between particles or precipitation in water, probably due to its low density (ca. 1.3 g/cm^3^), repulsion between surface charges (end group COO^−^), and the glassy state of the PI particles.

**Figure 2:**
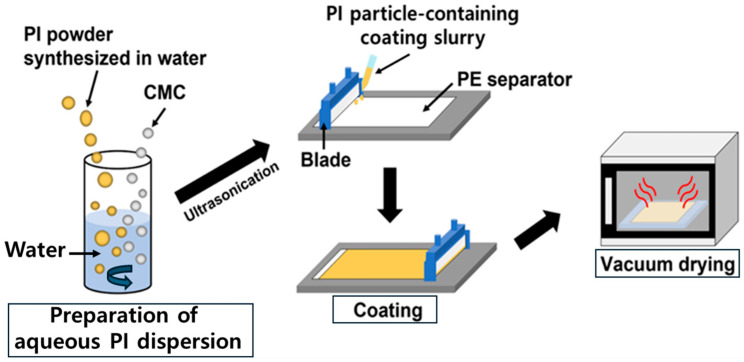
Schematic illustration of PI-PE separator preparation.

Figure 2 shows that the PI-coated PE (PI-PE) separators were prepared using water as a dispersant. After the PI powder and carboxymethyl cellulose (CMC) (as a binder) were placed in water, the resultant dispersion was subjected to ultrasonication at room temperature. The prepared slurries were coated onto one side of a Bare PE separator using a simple doctor blade coating process. The PI-PE separators were dried at room temperature and then at 50 °C in a vacuum. This process was eco-friendly and simple, in contrast to the conventional preparation of polymer-coated separators using expensive, toxic organic solvents and much higher drying temperatures.

Most inorganic or polymer particle coatings on polyolefin separators employ surfactants (e.g., disodium laureth sulfosuccinate, DLSS) or surface primers (e.g., polydopamine) in addition to binders. In this work, however, CMC was used solely as a binder and without any additional surfactants or surface primers. The success of our simple-component slurry coating might be due to the combination of strong CMC-PI adsorption (due to hydrogen bonding) and ‘mechanical interlocking’ imparted by the pores/roughness of the PE separator. In part, CMC might have provided rheology-driven continuous film formation on a non-wetting PE substrate [29].

The concentration of PI particles in the coating slurries was varied from 10 to 40 wt% while the CMC concentration was fixed at 0.5 wt%. When the coating slurry contained 10 wt% of PI particles, uniform coating could not be obtained. In addition, at 40 wt% PI particles, homogeneous dispersion was difficult. In a concentration range of 15–30 wt%, uniform coatings were obtained and showed low thermal shrinkage less than 5% and good wettability to a liquid electrolyte (1 M LiPF_6_ in ethylene carbonate/diethylene carbonate (EC/DEC), 1/1, *v*/*v*). Based on these results, as well as the ease and cost efficiency of the coating process, the formulation with the lowest polyimide content (15 wt%) was selected for further study. The contents of the CMC binder were adjusted to 0.1 wt%, 0.3 wt%, and 0.5 wt% to prepare PI-PE(0.1CMC), PI-PE(0.3CMC), and PI-PE(0.5CMC) separators, respectively.

### 3.2. Morphology and Thermal and Mechanical Properties

**Figure 3:**
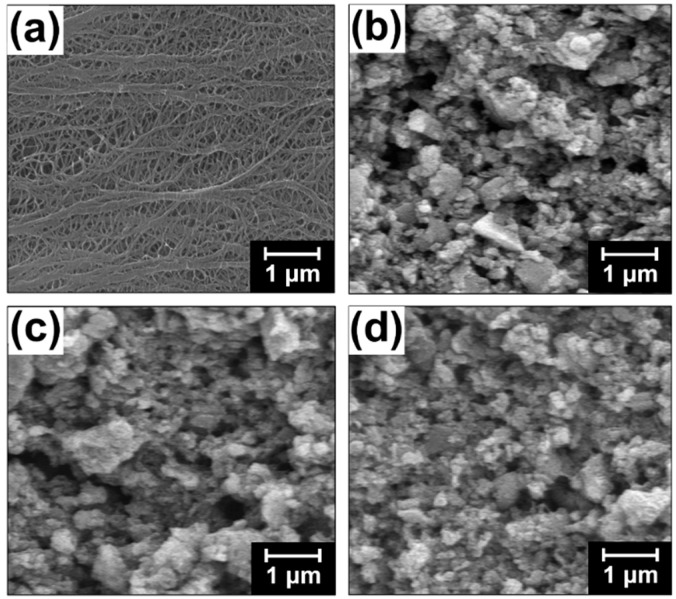
SEM images of the top surfaces of (**a**) Bare PE, (**b**) PI-PE(0.1CMC), (**c**) PI-PE(0.3CMC), and (**d**) PI-PE(0.5CMC) separators.

The surface morphologies of the Bare PE and PI-PE separators were observed by FE-SEM (Figure 3). While the Bare PE separator showed an interconnected pore structure, the PI-PE separators were covered by a layer of PI particles attached by CMC binder. The thickness of the Bare PE separator and PI-PE separators was measured from cross-sectional FE-SEM images (Figure S2). The thickness of the Bare PE separator was 12 μm (Figure S2a), while the PI-PE(0.1CMC), PI-PE(0.3CMC), and PI-PE(0.5CMC) separators had coating layers of 2.4 μm, 2.4 μm, and 2.5 μm thickness, respectively (Figure S2b–d). A relatively uniform coating layer was formed regardless of the CMC content.

**Figure 4:**
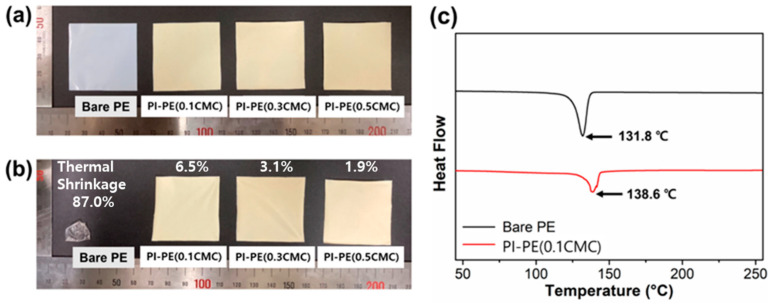
Photographs of Bare PE and PI-PE separators (**a**) at room temperature (25 °C) and (**b**) after exposure to 140 °C for 1 h. (**c**) DSC curves of Bare PE and PI-PE (0.1CMC) separators.

To evaluate the thermal stability of the Bare PE and PI-PE separators, their thermal shrinkage was investigated. As shown in Figure 4a,b, the Bare PE separator showed serious thermal shrinkage (87.0%) after exposure to 140 °C for 1 h due to its intrinsically poor thermal stability. The PI-PE separator exhibited a dramatic reduction in thermal shrinkage from 87.0% (Bare PE) to 6.5% or lower, demonstrating the outstanding thermal stabilization effect of the PI coating layer. The thermal shrinkage (6.5%) is lower than those of the reported polymer-particle-coated polyolefin separators (Table S1). This improvement in our work is mainly attributed to the intrinsically high thermal resistance and dimensional stability of PI, which effectively suppresses the heat-induced contraction of the PE substrate. The PI particles form a mechanically robust and thermally stable surface layer that constrains the deformation of the underlying PE matrix during heating. In addition, the interfacial adhesion between the PI coating and the PE surface contributes to the structural integrity of the separator, preventing pore collapse or melting at elevated temperatures. Such a remarkable reduction in shrinkage ensures the maintenance of mechanical integrity and electrical insulation under thermal abuse conditions, thereby significantly enhancing the safety of lithium-ion batteries. Figure 4c shows the DSC curves of the Bare PE and PI-PE(0.1CMC) separators. The Bare PE separator exhibited an endothermic peak in accordance with the melting point of PE at 131.8 °C. The DSC curve of the PI-PE(0.1CMC) separator showed an endothermic peak at 138.6 °C. This result implies that the PI-PE separator can shut down to prevent thermal runaway when the separator is exposed to high temperature above 140 °C. Figure S3 shows FE-SEM images of the Bare PE and PI-PE separators after exposure to 140 °C for 1 h.

**Figure 5:**
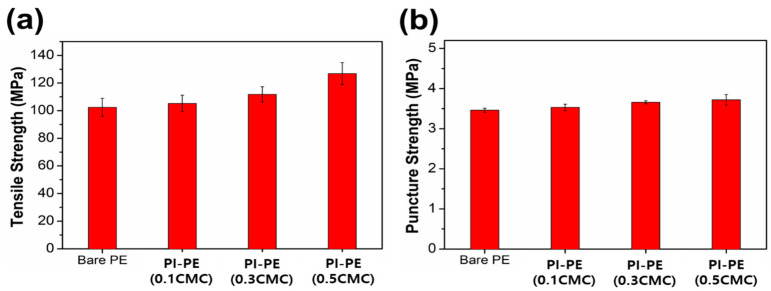
(**a**) Tensile strength and (**b**) puncture strength of Bare PE and PI-PE separators.

The mechanical strength of the separator plays an important role in suppressing the formation of lithium dendrites and other undesired impurities [30]. The tensile strength and the puncture strength of Bare PE and PI-PE separators were measured and are shown in Figure 5. Tensile strength values of the PI-PE(0.1CMC), PI-PE(0.3CMC), and PI-PE(0.5CMC) separators (105.1, 111.8, and 126.8 MPa, respectively) were higher than that of Bare PE separator (102.4 MPa). The tensile strength increased with increasing CMC content, probably because of the stronger binding effect. According to the ASTM standard [31], which specifies a minimum tensile strength of 98.06 MPa for separators, the present separators satisfied this requirement. On the other hand, PI-PE(0.1CMC), PI-PE(0.3CMC), and PI-PE(0.5CMC) showed higher puncture strength (3.53, 3.66, and 3.72 MPa, respectively) than that of the Bare PE separator (3.46 MPa). These results confirm that the PI particle coating can enhance the mechanical strength of a PE separator. These results also imply that the integrity of the separators can be maintained during cell assembly, and an internal short circuit caused by lithium dendrites or other impurities can be effectively prevented.

### 3.3. Wetting Property and Ionic Conductivity

**Figure 6:**
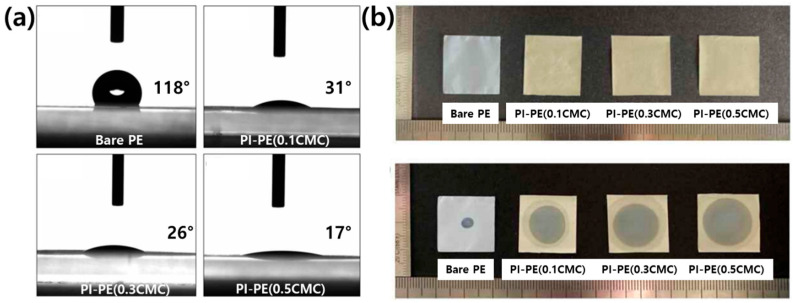
(**a**) Water contact angle images of the separators and (**b**) photographs of a liquid electrolyte droplet (EC/DEC 1:1 *v*/*v* with 1 M LiPF_6_) at 25 °C, taken before (**top**) and 5 min after (**bottom**) deposition onto each separator.

Proper wetting of a separator is helpful for cycle performance of LIBs because the separator is soaked with liquid electrolyte. The wettability of the present separators was characterized by a water contact angle measurement and a liquid electrolyte droplet test. Figure 6a shows that the water contact angle of the Bare PE separator was 118°, which drastically decreased to 31° on the PI-PE(0.1CMC) separator. The liquid electrolyte droplet test (Figure 6b) used a liquid electrolyte for commercial LIBs (EC/DEC (1:1, *v*/*v*) containing 1 M LiPF_6_). The electrolyte droplet placed on the Bare PE separator did not spread significantly, indicating the low wettability of the separator. In contrast, the droplets on the PI-PE separators were rapidly absorbed, and their spread area became much larger. These observations demonstrate that the coated separator has markedly improved electrolyte wettability and uptake. This improvement is likely due to the polar surface chemistry introduced by the PI particles (exposed imide carbonyls and terminal carboxyl/amino groups) together with the hydrophilic, anionic CMC binder, which increases the surface energy and promotes favorable interactions (dipole–dipole/hydrogen bonding) with the EC/DEC-based electrolyte.

The electrolyte uptake, porosity, and air permeability were tested to further characterize the porous structure of the separators, and the results are summarized in Table 1 and Table S1. The electrolyte uptake of the PI-PE separators increased markedly with the CMC binder content, from 91.7% at 0.1 wt% to 144.2% at 0.5 wt%, compared with 63.0% for the Bare PE separator. This result is consistent with the results of the water contact angle measurement and liquid electrolyte droplet test. This improvement can be attributed to the enhanced surface polarity and porosity introduced by the hydrophilic CMC binder. The CMC molecules, containing abundant hydroxyl and carboxyl groups, promote electrolyte wetting and facilitate liquid penetration into the separator pores. Moreover, increasing the binder content enhances the interparticle connectivity and coating uniformity, forming micro-channels that can hold more electrolyte. The increased electrolyte uptake may contribute to stable electrolyte distribution and improved interfacial contact between the separator and electrodes during electrochemical operation. However, an excessive binder content may lead to thicker coatings and possible pore blockage, so an optimal CMC ratio is required to balance electrolyte absorption and ion transport.

The porosity of the separators was tested by an *n*-butanol absorption method (Table 1). Since the character of *n*-butanol is similar to that of the commercial liquid electrolyte, the volume occupied by *n*-butanol after soaking is equal to the porosity of the separators. The measured porosity slightly decreased from 44.0% (Bare PE) to 38.3% (PI-PE(0.5CMC)) with increasing CMC content. The Gurley value of the separators increased progressively with higher CMC binder content (Table 1), from 173.2 s/100 mL for PI-PE(0.1CMC) to 219.0 s/100 mL for PI-PE(0.5CMC), compared with 135.6 s/100 mL for the Bare PE separator. The higher Gurley value indicates reduced air permeability, implying that the coating layer becomes denser as the amount of CMC increases. This behavior can be attributed to the improved interconnection among PI particles and the partial filling of surface pores by the CMC binder, which might diminish pore size and increase the tortuosity of diffusion pathways. Together with the higher electrolyte uptake but lower ionic conductivity, these data suggest that the hydrophilic binder improves affinity to the liquid while simultaneously constricting ion-transport pathways. Although excessive binder content may hinder electrolyte penetration or increase ionic resistance, the observed values remain within an acceptable range for lithium-ion battery separators: they were still lower than those of commercial separators [32].

**Figure 7:**
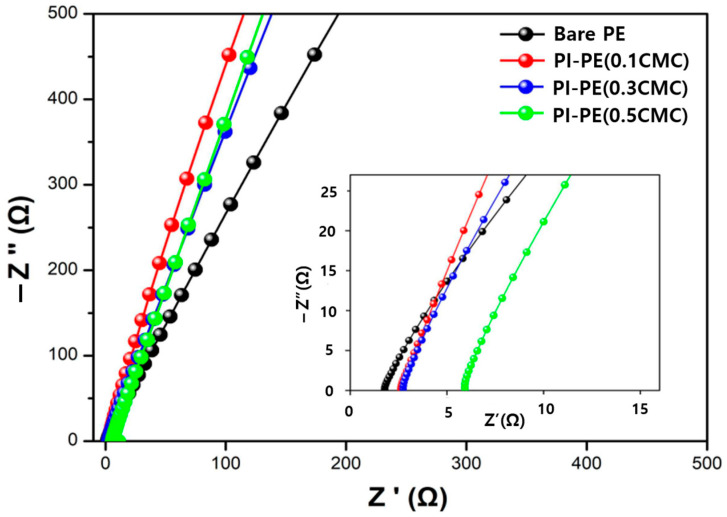
Nyquist plots of the cells (SS/separator/SS) for a liquid electrolyte-soaked Bare PE separator and a PI-PE(0.1CMC) separator at room temperature (25 °C).

The effective ionic conductivity (σ_eff_) of a separator soaked with liquid electrolyte is governed not only by the mobility and concentration of lithium ions in the electrolyte but also by the separator’s microstructure (porosity, tortuosity, and constriction) and by electrolyte uptake/wettability (which are influenced by viscosity and capillary infiltration). The σ_eff_ was evaluated by electrochemical impedance spectroscopy (EIS) using SS|separator|SS blocking cells. Figure 7 shows the Nyquist plots, where the *x*-axis represents the real impedance Z′, and the *y*-axis is the negative imaginary impedance −Z″. In this configuration, the high-frequency intercept on the real axis corresponds to the bulk resistance *R_b_*, from which the σ_eff_ was calculated. The σ_eff_ of the Bare PE, PI–PE(0.1CMC), PI-PE(0.3CMC) and PI-PE(0.5CMC) separators were calculated to be 1.191 mS cm^−1^ (*R_b_* = 1.78 Ω), 0.978 mS cm^−1^ (*R_b_* = 2.62 Ω), 0.938 mS cm^−1^ (*R_b_* = 2.73 Ω), and 0.432 mS cm^−1^ (*R_b_* = 5.93 Ω), respectively (Table 1). Although the electrolyte uptake of the PI-PE separators increased with increasing CMC content, σ_eff_ decreased from 0.978 mS cm^−1^ at 0.1 wt% to 0.432 mS cm^−1^ at 0.5 wt%, compared with 1.191 mS cm^−1^ for the Bare PE separator. This behavior can be explained by the structural densification of the coating layer caused by excessive binder addition. As the CMC content increases, the hydrophilic binder fills interparticle voids and partially blocks the separator pores, as indicated by the higher Gurley values. While moderate CMC levels enhance electrolyte wettability and retention through the formation of hydrophilic functional groups (–OH and –COOH), excessive binder results in reduced pore connectivity and increased tortuosity, which hinder ion transport through the separator.

Although the σ_eff_ slightly decreased after PI coating, the PI-PE(0.1CMC) separator maintained σ_eff_ comparable to those of previously reported polymer-coated polyolefin separators (Table S1) [18,19,20,21,22,23,24,25,26]. As noted above, the PI coating improves electrolyte wettability/uptake, but the added coating thickness (and slightly reduced air permeability/tortuosity effect) increases the bulk resistance, leading to a slight decrease in σ_eff_. However, because *R_b_* and σ_eff_ are sensitive to post-assembly soak time, stack pressure, temperature, and electrolyte formulation, we consider σ_eff_ of Bare PE and PI-PE(0.1CMC) to be comparable within experimental uncertainty [33,34]. Further, improved electrolyte wettability can facilitate intimate interfacial contact between the separator and electrode surface, thereby promoting homogeneous electrolyte distribution and stable Li-ion transport during charge–discharge operation. This is also consistent with the full-cell results, since σ_eff_ measured in SS|separator|SS blocking cells primarily reflects ion transport through the separator, whereas the charge–discharge capacity is also influenced by electrolyte/electrode interfacial contact, interfacial resistance, and lithiation/delithiation kinetics in the LiCoO_2_ electrode.

### 3.4. Electrochemical Cellular Performance

**Figure 8:**
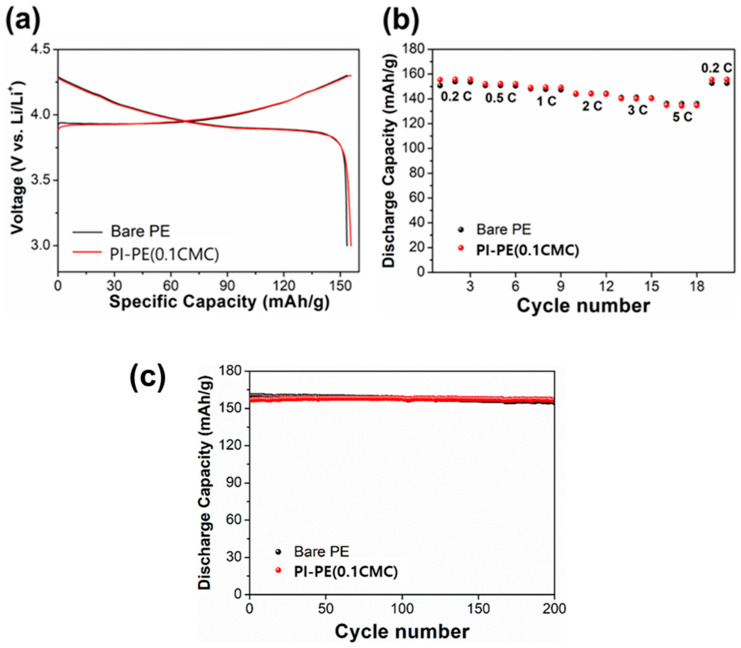
(**a**) Charge–discharge profiles, (**b**) rate capabilities, and (**c**) cycle performance of the cells (LiCoO_2_/separator/Li) assembled with Bare PE and PI-PE(0.1CMC) separators at room temperature (25 °C).

The electrochemical performance of the coin cells assembled with Bare PE and PI-PE(0.1CMC) separators was evaluated by charge–discharge property. The discharge capacity under constant current after the first charge and discharge of the cell was measured, and the initial reversible capacity of the cell was determined. When the cell in the initial charge state was discharged with a constant current, the voltage of the cell decreased. When the voltage decrease was less than a certain voltage, the discharge stopped, and the capacity was determined from the amount discharged during that time. Figure 8a shows charge–discharge curves of LiCoO_2_/separator/Li half-cell with Bare PE and PI-PE(0.1CMC) separators at a constant current density of 0.2 C. The half-cell with Bare PE separator had a charge capacity of 153.8 mAh/g and a maximum discharge capacity of 153.5 mAh/g (Coulombic efficiency = 99.8%). The half-cell with the PI-PE(0.1CMC) separator had a charge capacity of 155.7 mAh/g and a maximum discharge capacity of 155.7 mAh/g (Coulombic efficiency = 100.0%). These results indicate that the PI-PE(0.1CMC) separator maintained electrochemical behavior comparable to that of the Bare PE separator. In other words, the slightly lower σ_eff_ of PI-PE(0.1CMC) did not translate into a lower discharge capacity, most likely because the improved electrolyte wettability and stable separator/electrode contact compensated for the modest increase in bulk resistance at 0.2 C.

The rate capabilities of the half-cells assembled with the separators were evaluated at various charge–discharge rates (Figure 8b). The cells were charged within 3.0–4.3 V at a constant current density of 0.2 C and discharged at 0.2, 0.5, 1, 2, 3, and 5 C. Since fast charge–discharge is crucial, maintaining high capacity at high C-rates is important. As shown in Figure 8b, the half-cell with the PI-PE(0.1CMC) separator exhibited discharge capacities comparable to those with the Bare PE separator across all current densities, delivering 86.9% capacity retention relative to the initial discharge capacity at 5 C. Overall, the PI-PE(0.1CMC) separator exhibited rate capability comparable to that of the Bare PE separator over the investigated current-density range. The improved electrolyte wettability may contribute to stable electrolyte distribution during charge–discharge operation.

Figure 8c shows the cycle performance of LiCoO_2_/separator/Li half-cells with Bare PE and PI-PE(0.1CMC) separators under constant-current cycling between 3.0 and 4.3 V, at a charge–discharge rate of 1 C after a 0.1 C formation cycle. The PI-PE(0.1CMC) cell exhibited slightly lower initial discharge capacity (158.3 mAh/g) compared with the Bare PE cell (160.1 mAh/g). However, after 200 cycles, it retained 156.9 mAh/g (99.1%), surpassing the Bare PE cell (155.0 mAh/g, 96.8%). These results indicate that the PI-PE(0.1CMC) separator can maintain comparable durability during repeated cycling. Thus, PI particle coating not only enhances separator thermal stability but also maintains comparable electrochemical performance when applied in a cell.

## 4. Conclusions

A powder-type PI was synthesized using water as a solvent, and then PI-particle-containing coating slurries were prepared in an aqueous dispersion medium. The coating slurries were applied on a PE separator to obtain the PI-PE separators. The PI-PE separators exhibited markedly low thermal shrinkage (≤6.5%) at 140 °C for 1 h, whereas the Bare PE sample underwent severe shrinkage (87.0%) under the same conditions. Tensile strengths and puncture strengths of the PI-PE separators were higher than those of the Bare PE sample. Given that previously reported polymer-coated polyolefin separators exhibit σ_eff_ typically below ~0.87 mS cm^−1^, the PI-PE(0.1CMC) separator demonstrates an improved σ_eff_ (0.98 mS cm^−1^). The electrochemical properties of coin cells assembled with the PI-PE(0.1CMC) separator were evaluated by charge–discharge property. It was confirmed that LiCoO_2_/PI-PE separator/Li half-cells showed battery performance similar with Bare PE half-cells. This work provides insights into simple, eco-friendly preparation of a separator with excellent thermal stability, σ_eff_ and acceptable electrochemical performance.

## Figures and Tables

**Table 1:** Properties of Bare PE and PI-PE separators.

Separator	Thickness (μm)	Electrolyte Uptake (%)	Porosity (%)	Gurley Value (s/100 mL)	σ_eff_ (mS cm^−1^)
Bare PE	12	63.0	44.0	135.6	1.191
PI-PE(0.1CMC)	14.4	91.7	43.1	173.2	0.978
PI-PE(0.3CMC)	14.4	139.1	41.7	180.3	0.938
PI-PE(0.5CMC)	14.5	144.2	38.3	219.0	0.432

## Data Availability

Data are contained within the article and Supplementary Materials. Further inquiries can be directed to the corresponding authors.

## Supplementary Materials

The following supporting information can be downloaded at: https://www.mdpi.com/article/10.3390/polym18151871/s1, Scheme S1: Synthesis of PMDA-ODA PI in Water; Figure S1: Photographs of dispersions of SiO_2_, Al_2_O_3_, and PI powders in water at room temperature (a) before and (b) after storing for 24 h; Figure S2: SEM images of cross-section of (a) Bare PE separator, (b) PI-PE(0.1CMC) separator, (c) PI-PE(0.3CMC) separator, and (d) PI-PE(0.5CMC) separator; Figure S3: SEM images of the separators after exposure to 140 °C for 1 h of (a) top surface and (b) cross-section of Bare-PE separator, and (c) top surface and (d) cross-section of PI-PE(0.1CMC) separator; Table S1: Comparison of Properties of Polymer-Coated Polyolefin Separators.

## Abbreviations

The following abbreviations are used in this manuscript:

PI	Polyimide
PE	Polyethylene
PI-PE	PI-particle coated PE
LIB	Lithium-ion battery
PMDA	Pyromellitic dianhydride
ODA	4,4′-oxydianiline
CMC	Carboxymethyl cellulose
EC	Ethylene carbonate
DEC	Diethylene carbonate
DSC	Differential scanning calorimetry
FT-IR	Fourier transform infrared
FE-SEM	Field emission scanning electron microscope

## References

[1] Arora P., Zhang Z. (2004). Battery separators. Chem. Rev..

[2] Goodenough J.B., Park K.S. (2013). The Li-ion rechargeable battery: A perspective. J. Am. Chem. Soc..

[3] Zhang S.S. (2007). A review on the separators of liquid electrolyte Li-ion batteries. J. Power Sources.

[4] Tarascon J.M., Armand M. (2001). Issues and challenges facing rechargeable lithium batteries. Nature.

[5] Luiso S., Fedkiw P. (2020). Lithium-ion battery separators: Recent developments and state of the art. Curr. Opin. Electrochem..

[6] Wang F., Ke X., Shen K., Zhu L., Yuan C. (2022). A critical review on materials and fabrications of thermally stable separators for lithium-ion batteries. Adv. Mater. Technol..

[7] Ding Y., Jiang Y., Zeng C., Jin H. (2024). Recent progress of advanced separators for Li-ion batteries. J. Mater. Sci..

[8] Liu Z., Jiang Y., Hu Q., Guo S., Yu L., Li Q., Liu Q., Hu X. (2021). Safer lithium-ion batteries from the separator aspect: Development and future perspectives. Energy Environ. Mater..

[9] Parikh D., Jafta C.J., Thapaliya B.P., Sharma J., Meyer H.M., Silkowski C., Li J. (2021). Al_2_O_3_/TiO_2_ coated separators: Roll-to-roll processing and implications for improved battery safety and performance. J. Power Sources.

[10] Zhang H., Sheng L., Bai Y., Song S., Liu G., Xue H., Wang T., Huang X., He J. (2020). Amino-functionalized Al_2_O_3_ particles coating separator with excellent lithium-ion transport properties for high powder density lithium-ion batteries. Adv. Eng. Mater..

[11] Jiang X., Zhu X., Ai X., Yang H., Cao Y. (2017). Novel ceramic-grafted separator with highly thermal stability for safe lithium-ion batteries. ACS Appl. Mater. Interfaces.

[12] Yan L., Wang H., Sun Z., Wang L., Li C., Xue S., Liu Q. (2024). Enhanced performance of lithium-ion batteries based on a polyethylenimine-grafted SiO_2_-modified polypropylene separator. ACS Appl. Polym. Mater..

[13] Zhou D., He Y.-B., Liu R., Liu M., Du H., Li B., Cai Q., Yang Q.-H., Kang F. (2015). In situ synthesis of a hierarchical all-solid-state electrolyte based on nitrile materials for high-performance lithium-ion batteries. Adv. Energy Mater..

[14] Na W., Koh K.H., Lee A.S., Cho S., Ok B., Hwang S.-W., Lee J.H., Koo C.M. (2019). Binder-less chemical grafting of SiO_2_ nanoparticles onto polyethylene separators for lithium-ion batteries. J. Membr. Sci..

[15] Choi H., Lee B.S. (2022). Pilot-scale hybrid organic/inorganic coatings on a polyolefin separator to enhance dimensional stability for thermally stable long-life rechargeable batteries. Polymers.

[16] Peng L., Shen X., Dai J., Wang X., Zeng J., Huang B., Li H., Zhang P., Zhao J. (2019). Three-dimensional coating layer-modified polyolefin ceramic-coated separators to enhance the safety performance of lithium-ion batteries. J. Electrochem. Soc..

[17] Jang J., Oh J., Jeong H., Kang W., Jo C. (2020). A review of functional separators for lithium metal battery applications. Materials.

[18] Kim G., Noh J.H., Lee H., Shin J., Lee D. (2023). Roll-to-roll gravure coating of PVDF on a battery separator for the enhancement of thermal stability. Polymers.

[19] Song J., Ryou M.H., Son B., Lee J.N., Lee D.J., Lee Y.M., Choi J.W., Park J.K. (2012). Co-polyimide-coated polyethylene separators for enhanced thermal stability of lithium-ion batteries. Electrochim. Acta.

[20] Ding H., Ge J., Zhang T., He C., Zhang H., Ali B.M., Wang J. (2024). Thermally stable poly-aromatic solid electrolyte-coated polyethylene membrane as a high-performance lithium-ion battery separator. J. Power Sources.

[21] Luo X., Da X., Wu Y., Zhang D., Zhang Z., Yang F., Cao Y. (2025). A novel EVOH-coated polyethylene separator for high-performance lithium-ion batteries. Ind. Eng. Chem. Res..

[22] Lee Y., Ryou M.H., Seo M., Choi J.W., Lee Y.M. (2013). Effect of polydopamine surface coating on polyethylene separators as a function of their porosity for high-power Li-ion batteries. Electrochim. Acta.

[23] Hu S., Lin S., Tu Y., Hu J., Wu Y., Liu G., Li F., Yu F., Jiang T. (2016). Novel aramid nanofiber-coated polypropylene separators for lithium-ion batteries. J. Mater. Chem. A.

[24] Shi J.L., Fang L.F., Li H., Zhang H., Zhu B.K., Zhu L.P. (2013). Improved thermal and electrochemical performances of PMMA-modified PE separator skeleton prepared via dopamine-initiated ATRP for lithium-ion batteries. J. Membr. Sci..

[25] Ye W., Fan Z., Xue Z. (2024). Functionalized polypropylene separator coated with polyether/polyester blend for high-performance lithium metal batteries. Energy Mater..

[26] Jeong Y.B., Kim D.W. (2005). The role of an adhesive gel-forming polymer coated on a separator for rechargeable lithium metal polymer cells. Solid State Ion..

[27] Cho Y.J., Kim D.M., Song I.H., Choi J.Y., Jin S.W., Kim B.J., Jeong J.W., Jang C.E., Chu K., Chung C.M. (2018). An oligoimide particle as a Pickering emulsion stabilizer. Polymers.

[28] Song I.H., Kim D.M., Choi J.Y., Jin S.W., Nam K.N., Park H.J., Chung C.M. (2019). Polyimide-based PolyHIPEs prepared via Pickering high internal phase emulsions. Polymers.

[29] Bertin V., Snoeijer J.H., Raphaël E., Salez T. (2022). Enhanced dip coating on a soft substrate. Phys. Rev. Fluids.

[30] Lei Y., Xu L., Chan Q.N., Li A., Yuen A.C.Y., Yuan Y., Yeoh G.H., Wang W. (2024). Recent advances in separator design for lithium metal batteries without dendrite formation: Implications for electric vehicles. eTransportation.

[31] Li L., Duan Y. (2023). Engineering polymer-based porous membrane for sustainable lithium-ion battery separators. Polymers.

[32] Lagadec M.F., Zahn R., Wood V. (2019). Characterization and performance evaluation of lithium-ion battery separators. Nat. Energy.

[33] Li M., Zhang Z., Yin Y., Guo W., Bai Y., Zhang F., Zhao B., Shen F., Han X. (2020). Novel polyimide separator prepared with two porogens for safe lithium-ion batteries. ACS Appl. Mater. Interfaces.

[34] Li Y., Wang X., Liang J., Wu K., Xu L., Wang J. (2020). Design of a high-performance zeolite/polyimide composite separator for lithium-ion batteries. Polymers.

