# Supplementary material for: Eco-Friendly Preparation of a Polyimide/Polyethylene Composite Separator and Its Application in Lithium-Ion Batteries
Source: Polymers (Basel). 2026 Jul 30;18(15):1871. doi: 10.3390/polym18151871 (PMC13467858; doi:10.3390/polym18151871)
Supplement: Supplementary file 1 [file polymers-18-01871-s001.zip › polymers-4446251-supplementary.pdf]

Supplementary Material

Eco-Friendly Preparation of Polyimide/Polyethylene Composite Separator and Its Application in Lithium-Ion Batteries

Hyun-Soo An [1,†], Yun-Je Choi [1,†], Dam-Bi Kim [1], Yasaswini Oruganti [1], Dae-Woon Lim [1], Seungwon Song [2,*], Woojun Choi [1,*], Chan-Moon Chung [1,*]

[1] Department of Chemistry, Yonsei University, Wonju 26493, Republic of Korea

[2] Xerabrid Corporation, Hwaseong 18469, Republic of Korea

[*] Corresponding author: cmchung@yonsei.ac.kr

†These authors contributed equally to this work.

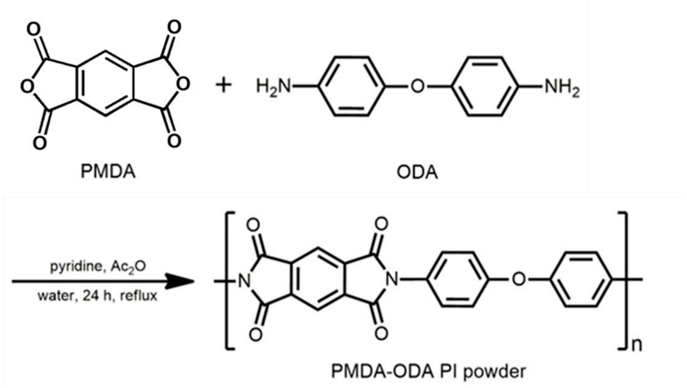

Scheme S1. Synthesis of PMDA-ODA PI in Water

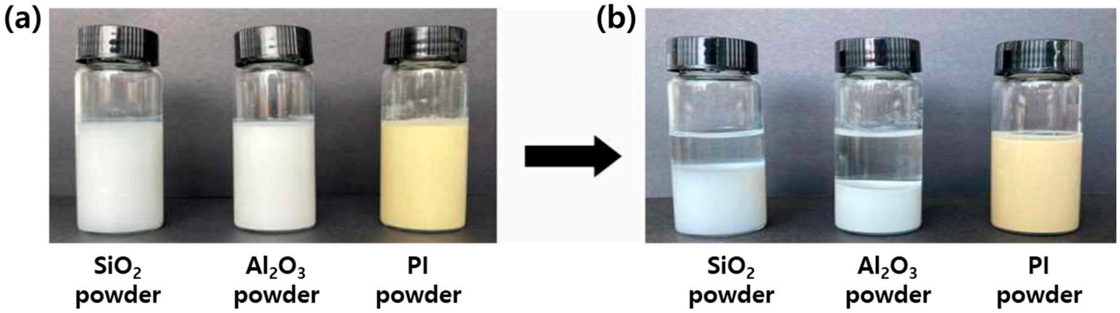

Figure S1. Photographs of dispersions of SiO_2, Al_2O_3, and PI powders in water at room temperature (a) before and (b) after storing for 24 h.

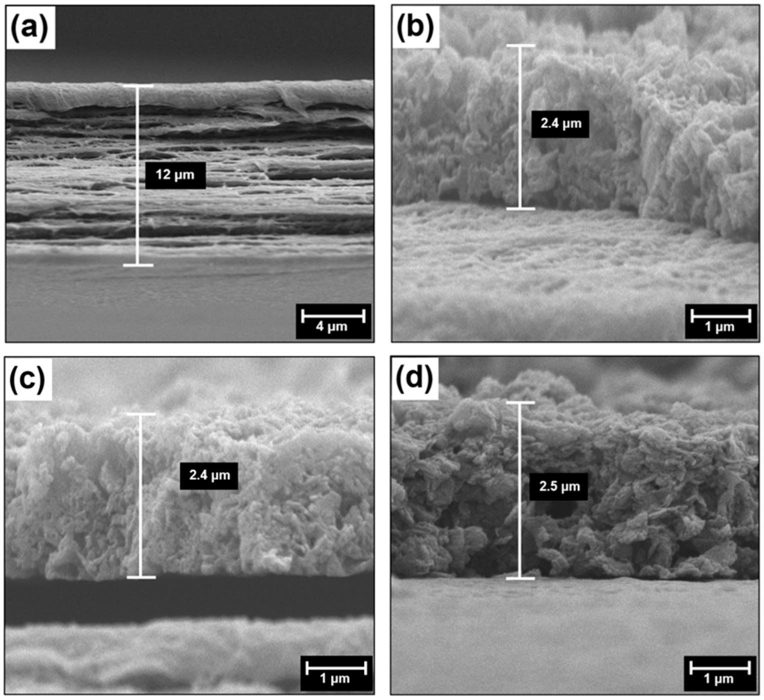

Figure S2. SEM images of cross-section of (a) Bare PE separator, (b) PI-PE(0.1CMC) separator, (c) PI-PE(0.3CMC) separator, and (d) PI-PE(0.5CMC) separator.

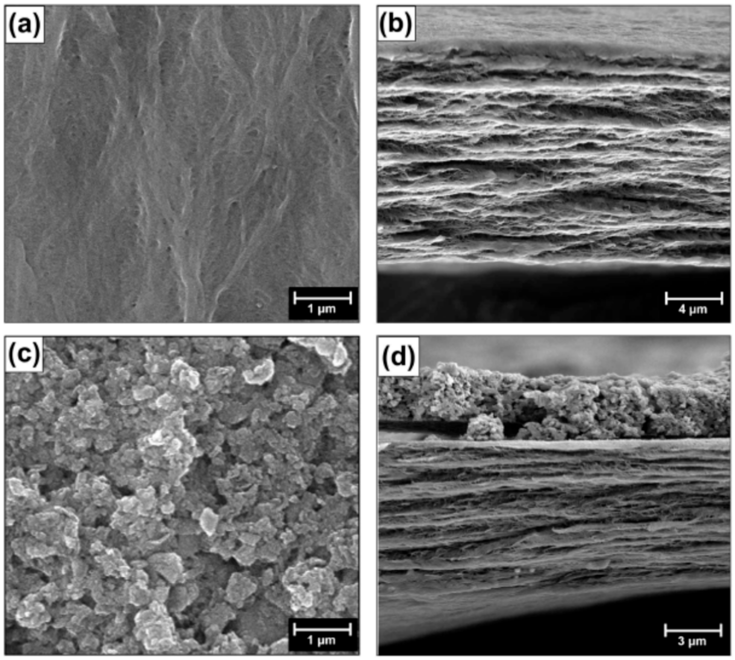

Figure S3. SEM images of the separators after exposure to 140 °C for 1 h of (a) top surface and (b) cross-section of Bare-PE separator, and (c) top surface and (d) cross-section of PI-PE(0.1CMC) separator.

Table S1. Comparison of Properties of Polymer-Coated Polyolefin Separators

Separator	Thermal shrinkage (%)	Thickness (μm)	Electrolyte uptake (%)	Porosity (%)	Gurley value (s/100 mL)	σ_{eff} (mS cm^{-1})
This work (PI-PE(0.1CMC))	6.5	14.4	91.7	43.1	173.2	0.978
Ref. 18	~15	-	-	-	-	-
Ref. 19	10–13	>20	109	-	152–208	0.243
Ref. 20	~30	~12	80–130	40–50	-	0.238–0.355
Ref. 21	~40	-	>200	40–50	>350	-
Ref. 22	-	16	169–202	41–47	187–266	0.669–0.874
Ref. 23	~4	-	130	40–50	>250	0.69–0.76
Ref. 24	>10	-	>180	31–58	>100	1.19
Ref. 25	9	-	-	-	-	>0.6
Ref. 26	-	-	84–90	-	-	0.77–1.0

"–" indicates that the value was not reported in the referenced paper.
